# Integrating Exposome into Lifecourse Understanding of Cognitive Ageing and Dementia: Current Evidence, Methodological Challenges, and Future Directions

**DOI:** 10.3390/ijerph22060815

**Published:** 2025-05-22

**Authors:** Jessica Gong, Paola Zaninotto

**Affiliations:** 1 Department of Epidemiology and Public Health, University College London, London WC1E 7HB, UK; p.zaninotto@ucl.ac.uk; 2 George Institute for Global Health, Imperial College London, London W12 7RZ, UK; 3 Department of Neurobiology, Care Sciences & Society, Karolinska Institutet, 171 77 Stockholm, Sweden

## 1. Introduction

Cognitive decline and dementia represent some of the most pressing challenges to global public health, especially amid rapidly ageing populations worldwide [1,2]. Accumulating evidence on the risk factors for dementia challenges the traditional genetic and biomedical frameworks in explaining the heterogeneity in dementia risk, highlighting that certain lifestyle and environmental factors play more crucial roles in contributing to the risk of dementia [2,3]. For example, individuals carrying the APOE ε4 allele—a well-established genetic risk factor for Alzheimer’s disease—do not universally develop dementia [4], underscoring the limitations of framing dementia aetiology through a purely genetic lens.

Alzheimer’s disease and related dementias (ADRD) are increasingly recognized as systemic disorders with multifactorial aetiology [2,3], demanding a shift from isolated risk factor analyses to integrative models that capture synergistic effects. The 2024 update of the Lancet Commission on ADRD identified 14 potentially modifiable risk factors for dementia, including the following: lower educational attainment, hearing impairment, elevated low-density lipoprotein (LDL) cholesterol levels, traumatic brain injury, low physical activity, diabetes mellitus, smoking, hypertension, obesity, excessive alcohol intake, social isolation, air pollution, and visual impairment [2]. Importantly, the majority of these risk factors demonstrate strong links to broader environmental conditions and socioeconomic determinants of health [2].

To better understand how these external influences translate into biological processes that drive neurodegeneration, researchers have increasingly turned to multi-omics approaches. Advances in multi-omics technologies (e.g., genomics [5,6], proteomics [7,8,9,10,11,12], metabolomics [13,14]) have facilitated the omics-wide identification of novel biomarkers and biological signatures linked to ADRD. These breakthroughs have expanded our understanding of disease mechanisms, yet they represent only part of a larger, more complex picture. A growing body of research emphasizes the critical role of environmental exposures across the lifespan, interacting dynamically with genetic and molecular mechanisms and contributing to the acceleration of cognitive ageing [15,16]. Innovations in environmental monitoring—such as the geospatial tracking of pollution and climate, wearable devices quantifying direct pollution exposure, and high-resolution biomonitoring of chemical toxins—now enable the precise measurement of external stressors [17]. Recognising the need to systematically capture the full breadth of environmental influences, recent research has expanded into the exposome paradigm, a comprehensive framework that integrates cumulative environmental exposures (e.g., physical, chemical, social, lifestyle) and their biological interactions to decode disease aetiology [18]. When examined through the lens of exposome science—which encompasses the totality of human environmental exposures from prenatal development through adulthood—these risk factors emerge as interconnected components of a complex biological–environmental system [19,20]. Rather than functioning independently, these elements interact in dynamic and often synergistic ways. This exposomic perspective can fundamentally transform our thinking around dementia prevention and neurological mechanisms [21,22]. It moves beyond conventional models focused primarily on individual lifestyle choices to recognize how structural factors—including macro-level determinants such as educational opportunities, urban planning decisions, environmental regulations, and social support systems—collectively shape population-level dementia risk trajectories [23,24].

A landmark exposome-wide association study (XWAS) using the UK Biobank data recently quantified the contribution of exposomes, age, sex, and polygenic risk scores to 22 major diseases [25]. For dementia, particularly vascular dementia, environmental exposures accounted for a substantial proportion of the risk, although smaller than the contributions from age, sex, or genetics [25]. However, exposomes represent the most modifiable component of dementia risk and thus offer some of the clearest targets for prevention. Despite this potential, research remains disproportionately focused on late-life biomarkers, neglecting the long-term exposures accumulating since early life. Longitudinal epidemiological studies reveal that dementia risk begins accumulating as early as childhood [26], or even with transgenerational effect [26,27], underpinned by factors such as educational access, nutritional deficiencies, and socioeconomic disparities [2,28].

Effectively, an exposomic approach to studying health outcomes requires a comprehensive, discovery-driven framework that addresses the following three key research needs: (1) comprehensive longitudinal exposure assessment; (2) improved measurement of physiological and biological responses; and (3) integration of multidisciplinary research [29]. This commentary explores these aspects with a focus on dementia and cognitive ageing, highlighting both methodological challenges and future research opportunities.

## 2. Methodological Challenges

The shift toward exposome-driven frameworks for understanding dementia risk comes with a plethora of methodological complexities. While advances in multi-omics and environmental monitoring technologies offer unprecedented opportunities to comprehensively map the interplay between biological, environmental, and social factors, several critical challenges hinder progress in disentangling causal pathways and translating findings into prevention strategies.

### 2.1. Multidimensional Data Complexity

Exposome research necessitates the integration of vast, heterogeneous datasets that encompass both external exposures and internal biological responses. This integration introduces several interrelated challenges.

First, measurement granularity poses a significant hurdle. While high-resolution environmental monitoring tools like wearable sensors and geospatial tracking systems generate terabytes of dynamic exposure data [30], inconsistencies in the spatial and temporal resolution across studies limit the comparability of datasets, thereby hindering cross-study validation and meta-analyses.

Second, the integration of multi-omics, multi-modal data with environmental variables demands robust computational pipelines to address technical noise, missing data, and batch effects [31]. For example, aligning metabolomic, proteomic, or genomic datasets with external exposure metrics requires harmonizing disparate data types while accounting for variability introduced by differing laboratory protocols, sampling intervals, or even sample type, which can obscure biologically meaningful signals.

Third, the high-dimensional nature of exposome data amplifies statistical challenges. Biomarker studies, for instance, may analyse over thousands of markers simultaneously, creating a “needle-in-a-haystack” scenario for identifying true associations. To address this, more researchers are employing machine learning methods (e.g., random forests, support vector machines, neural networks, gradient boosting), dimensionality reduction techniques (e.g., principal component analysis or latent variable models), and Bayesian frameworks that incorporate prior knowledge to improve signal detection [32,33]. These approaches can help disentangle complex, non-linear relationships and uncover latent patterns in high-dimensional exposome data. However, even with these advanced analytic approaches, the proliferation of variables continues to increase the risk of false discoveries, necessitating stringent corrections for multiple testing, such as using the Benjamini–Hochberg method to correct for false discovery rate [34]. However, overly conservative statistical approaches risk overlooking subtle yet critical interactions, such as low-dose chronic exposures or synergistic effects between multiple factors. Collectively, these challenges underscore the need for advanced computational frameworks and standardized protocols to manage the complexity of exposome data [24], while preserving its utility for dementia research.

### 2.2. Temporal and Lifecourse Dynamics

Dementia risk unfolds over decades, shaped by dynamic interactions between environmental exposures and biological processes across the lifespan. However, research often prioritizes late-life exposures, overlooking critical windows of vulnerability during prenatal, perinatal, and early childhood periods where neurodevelopment is especially vulnerable. This narrow focus introduces three interrelated methodological challenges.

First, the scarcity of longitudinal data limits insights into lifelong risk trajectories. Few cohort studies track individuals from early life to old age, and many cohorts often face attrition biases. Survival bias—where healthier individuals disproportionately remain in long-term studies—can skew risk profiles, masking the full impact of early-life adversities on subsequent cognitive decline [35]. Repeated biomarker measurements are essential to capture dynamic biological changes in response to both acute and chronic environmental exposures.

Second, time-varying interactions complicate causal inference [36], as exposures may exert delayed, nonlinear, or cumulative effects. For instance, childhood poverty could predispose individuals to midlife metabolic dysfunction and limited cognitive stimulation, both of which influence brain structure and stress reactivity, amplifying dementia risk decades later. Such lagged relationships challenge conventional statistical models, which often assume static or linear associations [36].

Third, retrospective exposure assessment introduces measurement inaccuracies. Reliance on self-reported childhood exposures—such as dietary habits or residential history—is prone to recall bias, as older adults may misremember details or conflate timelines [37]. Proxy informants or historical records are often incomplete or inconsistently available [38]. Even when objective biomarkers using frozen blood samples are used, gaps in historical environmental monitoring limit the precision of retrospective exposure reconstruction [39]. These challenges underscore the urgent need for lifecourse cohorts that integrate prospective environmental monitoring with longitudinal multi-omics profiling, enabling researchers to disentangle how the timing, duration, and interactions of exposures shape dementia risk across the lifespan.

### 2.3. Confounding and Reverse Causation

Environmental exposures do not act in isolation but are intricately entwined with socioeconomic, behavioural, and genetic factors, creating a web of analytical challenges that complicate efforts to isolate causal pathways in dementia research. Three central issues arise in this context.

First, residual confounding undermines attempts to disentangle environmental effects from correlated social determinants. While studies often adjust for confounders, many fail to account for their cumulative or time-varying impacts across the lifecourse.

Second, reverse causation introduces ambiguity in interpreting exposure–dementia associations [40,41]. Early neurodegenerative processes, such as the accumulation of amyloid-beta plaques years before clinical symptoms emerge [42], may subtly alter behaviour or lifestyle, thereby distorting exposure measurements. A person in the preclinical stages of Alzheimer’s disease might reduce outdoor physical activity due to apathy or motor changes, leading to artificially low pollution exposure readings that may be mistaken for protective effects, when in fact, they reflect disease-driven behavioural changes.

Third, gene–environment correlation (rGE) obscures causal inferences by linking genetic predispositions to environmental experiences [43]. This may sometimes create a scenario where genetic and environmental risks are conflated [43], making it difficult to discern whether outcomes arise from molecular mechanisms, external exposures, or their interplay. Twin studies have highlighted such dynamics, showing that genetic predispositions can influence exposure-prone behaviours [44], which in turn modify dementia risk trajectories.

These challenges collectively underscore the need for innovative study designs—such as sibling-matched cohorts, Mendelian randomization, lifecourse-adjusted models, and triangulating findings through methods like longitudinal cohort replication and quasi-experimental designs—to disentangle the genetic, environmental, and social threads in dementia research.

### 2.4. Equity and Generalizability Gaps

Exposome–dementia research holds promise for advancing prevention strategies, but methodological oversights risk perpetuating—or even exacerbating—health disparities if studies fail to account for marginalized populations [45]. The following three interrelated challenges threaten the equity and generalizability of the findings: First, sampling bias systematically excludes underrepresented groups from research. Most large-scale exposome studies disproportionately enrol high-income individuals and predominantly populations of Caucasian ancestry [46]. This neglects rural communities, low-resource settings, and racially diverse groups who face unique exposure profiles [45]. The similar pattern is observed in genomics studies [47]. Consequently, interventions derived from biased samples may fail to address the exposures most harmful to marginalized groups, widening global dementia inequities. Second, measurement inequity further compounds these gaps. Innovative tools like wearable sensors, smartphone-based GPS tracking, and real-time pollution monitors are often inaccessible in underserved communities due to cost, infrastructure limitations, or digital literacy barriers [48]. This creates “data deserts” that obscure localized risks and reinforce a Eurocentric evidence base. Lastly, ethical dilemmas arise when collecting granular environmental data in vulnerable populations. Linking precise geospatial tracking (e.g., GPS logs of daily movements) to health outcomes also raises privacy concerns [49], particularly for stigmatized groups such as undocumented immigrants or racial minorities already subject to surveillance.

These challenges highlight a plain reality: without intentional efforts to ensure equity is rooted in study design, exposome-dementia research risks validating interventions that benefit already privileged populations while leaving those most vulnerable to systemic environmental harms behind. Bridging these gaps demands community-engaged methodologies, equitable resource allocation for global monitoring tools, and ethical frameworks that prioritize data sovereignty and actionable redress for marginalized groups.

## 3. Future Directions and Innovations

### 3.1. Toward a Lifecourse and System-Level Exposome Framework

To address these gaps, the exposome framework must incorporate temporal and spatial dimensions, integrating lifecourse epidemiology to identify critical exposure windows (e.g., prenatal development, midlife) and cumulative risk patterns. For example, childhood lead exposure may be prime neuroinflammatory pathways, while midlife occupational stressors compound this risk, culminating in late-life cognitive decline [28,50]. Similarly, the dynamic interactions between environmental exposures (e.g., green space access) and individual resilience factors (e.g., cognitive reserve, social networks) must be mapped to reveal potentially protective pathways.

### 3.2. Technological Advances in Exposome Measurement

The growing yet fragmented evidence linking exposomes to dementia underscores the urgency for technological and methodological innovation. Advances in wearable sensors [51], digital biomarkers [52], and AI-driven exposure tracking offer transformative potential to address challenges in data harmonization and high-dimensional exposome data analysis. These tools enable the real-time, granular monitoring of environmental, lifestyle, and social exposures, while machine learning algorithms can disentangle complex interactions across heterogeneous datasets. Large-scale biobanks and cohort studies with longitudinal exposome data further provide a foundation for integrating multi-omics profiles to identify mechanistic pathways [53].

### 3.3. Innovative Index Generation and Profile Clustering

Using multi-omics biological data (e.g., genomics, epigenomics, proteomics) in combination with multi-modal neuroimaging (e.g., structural, functional, and diffusion MRI), more nuanced indices (i.e., brain age index) and ageing clocks (i.e., brain clock) [54,55] can be constructed to quantify deviations from healthy neurobiological ageing. For instance, a paper using proteomics data identified three distinct waves of brain ageing [51], underscored how integrative approaches can reveal temporally structured biological pathways underlying brain ageing, enabling the early detection of pathological trajectories and personalized interventions. These integrative models not only enhance predictive accuracy but also uncover mechanistic insights into age-related cognitive decline and neurodegenerative diseases. By leveraging machine learning and cross-modal data fusion, this approach helps determine personalized biomarkers for early intervention and tailored therapeutic strategies. Within the context of environmental exposure, metrics such as the Heat Index [56] and the Index of Multiple Environmental Deprivation (IMED) [57] can quantify cumulative stressors. When combined with multi-omics and neuroimaging-derived brain ageing indices, these tools enable a systems-level understanding of how external exposures accelerate or decelerate biological ageing [54]. This integrative approach uncovers critical gene/bio-environment interactions, offering new avenues for precision public health interventions. Nevertheless, while machine learning offers promise, its application in exposome research must be carefully validated to avoid overfitting or spurious correlations in high-dimensional data.

### 3.4. Interdisciplinary Collaboration for Holistic Insights

Progress also hinges on interdisciplinary collaboration bridging environmental health, neurology, computational biology, and social sciences. Such partnerships are critical to contextualize exposome–dementia links within broader societal structures and data harmonization, for example, exploring how socioeconomic disparities amplify toxic exposures or how cognitive reserve modifies risk. Social scientists can elucidate resilience factors, while computational biologists develop models to untangle gene–environment interactions and biological mechanisms. Concurrently, neurologists must refine dementia subtypes and cognitive assessments tied to specific exposures, ensuring prevention strategies are biologically grounded. Large research consortia, such as the International Human Exposome Network (IHEN) (https://humanexposome.net/, accessed on 9 May 2025), the Gateway Exposome Coordinating Center (https://gatewayexposome.org/, accessed on 9 May 2025), Exposome NL (https://exposome.nl/, accessed on 9 May 2025), and the EXPANSE consortium (https://expanseproject.eu/, accessed on 9 May 2025), among others [33], have systematically mapped and harmonized diverse exposome data across multiple cohorts. This effort ensures comparability and enhances cross-cohort and cross-national validity.

## 5. Conclusions

Adopting an exposome lens is indispensable for unravelling the multifactorial aetiology of dementia. While methodological hurdles persist, progress in technology, interdisciplinary collaborations, methodological developments and equity-focused data harmonization, as well as policy-driven action can bridge existing gaps. A global call to action is needed, prioritizing funding for longitudinal exposome studies, standardizing exposure metrics, and fostering inclusive cohorts that represent diverse populations. By centring equity in both research and practice, the field can move beyond fragmented evidence toward actionable strategies that promote cognitive resilience across the lifecourse.

We encourage researchers who use innovative research methods to capture the effects of exposomes on dementia and cognitive ageing to submit their work to the Special Issue of the *International Journal of Environmental Research and Public Health* on “Exposomic Approach to Dementia and Cognitive Ageing”.
